# Medicine and Dentistry

**Published:** 1887-01

**Authors:** 


### MEDICINE AND DENTISTRY.

The address of Dr. Kingsley, printed in this number, will undoubtedly call out protests from many quarters. In accepting it for publication, it will, of course, be understood that neither the editor nor the publishers are committed to its propositions. Indeed, a standing notice at the commencement of each number expressly states this of any accepted article. But we deem it the duty of a journal which aims to reflect the opinions and actions of any particular body of men to let both sides of any vital question be heard, provided it has two sides. The question of the relation of dentistry to medicine is by no means a novel one, for it has periodically convulsed some part of our number ever since the early days of Harris and Bond. The same arguments on both sides have been again and again urged, only to be met with the same counter-statements. It is time that the relative status should be determined, and there is no better way to accomplish this than through a temperate discussion of the matter, which shall assist every dentist in deciding for himself, and shall qualify him to give an intelligent vote and voice in settling the future policy of his profession.

It should be remembered that dentistry in America occupies an anomalous position. Whether wisely or not, and whether for the ultimate best interests of dentistry or not, the founders of our modern practice had no alternative, if educated and thoroughly equipped members were to be received, except in the establishment of segregated schools, and the conferring of a distinct diploma. This cut us off from medicine, for no one will for a moment imagine that the mother profession could acknowledge any diploma save her own, or recognize any school as competent to confer that diploma save regularly constituted medical ones, teaching the whole science of medicine. In no other country does this anomaly exist. In most of the European nations, until within a recent time, to become a fully recognized and approved dentist, a complete medical training was necessary. Now, in most of them, a special curriculum of dental study has been established, and the dentist recognized by medicine is not required to graduate in general surgery or obstetrics.

In America, the tendency is toward a higher education in dentistry, and since the peculiar course of some of our separate schools in cheapening the dental degree and conferring it upon doubtful candidates, foreigners especially, there has been a deepening feeling in favor of a degree that has not been, in a measure, tainted by unworthy men. Years ago, we drew upon ourself the censure of some of our separate schools, by the statement that, in our opinion, the tendency of the times was toward the education of dentists in medical schools with special dental departments. It was a simple, bald statement of what we believed to be a fact, whether of good or evil import. The course of the dentists in other countries in securing the establishment of dental professorships in the universities, and the sending of dental students to those institutions, has proved that we were correct.

In America we have arrived at the parting of the ways. Shall we, in the future, endeavor to draw yet closer the bond which unites us to the mother profession, or shall we strike out an independent course, cut ourselves adrift from medicine and burn our bridges behind us? It is time that a future policy be marked out, and we enter upon an intelligently resolved course of action. This can only be determined by calm and dispassionate debate. It is not the part of wisdom to appeal to prejudice, nor to allow our passions to dominate our action. The disputant who appeals to personal bias, or who harbors unworthy and ulterior motives, should be allowed no voice in our discussions. Hard words only provoke hard words in return. Our opponents are not half as bad men as we usually picture them.

It is not to be supposed that this is a question of the existence of our separate schools. Their immediate and unconditional abolition, even were it possible, would be a backward step. Neither is it the diploma of D. D. S. *versus* that of M. D. This question need not enter into the consideration of the case. In England, where the relations of dentistry and medicine are so close, the separate degree of Licentiate of Dental Surgery (L. D. S.) has been established, but it emanates from a medical source, and the qualified dentist is amenable to a medical tribunal. In Germany, the approbated dentist derives his authority to practice from a medical court. It is not, perhaps, generally known that, in one State of our Union—Alabama—long before the war, an enactment placed dentistry directly under the control of medicine, and gave to medical authorities the sole right to license dentists to practice. The gulf which separates the two is not now a wide one, and it may be bridged over or irreparably extended. The question to be decided is, whether we shall, by precipitate action, sever all ties, or endeavor to get into yet closer relationship with medicine. We cannot occupy the anomalous position of neither actually belonging to it or being separated from it.

There is one thing of which we are thoroughly convinced. This is no time for antagonizing the Medical Congress which is to meet in Washington this year. Every sentiment, whether of patriotism or professional pride, demands that factional feeling should be suppressed. There are dentists who, for reasons of their own, decline to take any part in the dental section of that meeting. That is a matter for their own judgment to decide. No man can impugn their motives, nor call in question their honesty and sincerity in such declination, and the few who have ventured to criticise the calm decisions of those who do not choose to join in the movement for the establishment of a dental section have usually been sternly rebuked by their own friends. Any factional course by those who are not identified with the section should be frowned down. No matter what may be thought by any one of the advisability of that meeting, it would be despicable to attempt to bring about a failure through seditious action. We do not mean to intimate that such a thing is contemplated, for we are certain that it is not. But the attempt at the present time to organize an International Dental Congress would, without doubt, be so interpreted, and hence we believe this to be a most unfortunate time for the agitation of that question. Let each do what he consistently can, and what his own sense of right prompts him to do for the success of the International Meeting of 1887, and after that it will be quite time enough to consider the propriety of a Dental Congress.

